# The Goal of Achieving High-Quality Surgical First-Line Therapy in Patients with Penile Cancer Is Important; However, Some Collective Efforts Are Still Required in Order to Reach It. Comment on Brassetti et al. Combined Reporting of Surgical Quality and Cancer Control after Surgical Treatment for Penile Tumors with Inguinal Lymph Node Dissection: The *Tetrafecta* Achievement. *Curr. Oncol.* 2023, *30*, 1882–1892

**DOI:** 10.3390/curroncol30040325

**Published:** 2023-04-19

**Authors:** Matthias May, Steffen Lebentrau, Ben Ayres, Maarten Albersen, Chris Protzel, Jad Chahoud, Oscar R. Brouwer, Curtis A. Pettaway, Lance C. Pagliaro, Andrea Necchi, Nick Watkin, Oliver W. Hakenberg, Philippe E. Spiess

**Affiliations:** 1 Department of Urology, St. Elisabeth-Clinic Straubing, Brothers of Mercy Hospital, 94315 Straubing, Germany; 2 Department of Urology, University of Magdeburg, 39106 Magdeburg, Germany; 3 Department of Urology, St George’s University Hospitals NHS, London SW17 0QT, UK; 4 Department of Urology, University Hospitals Leuven, 3000 Leuven, Belgium; 5 Department of Urology, Helios Clinics Schwerin, 19055 Schwerin, Germany; 6 Department of Genitourinary Oncology, H. Lee Moffitt Cancer Center, Tampa, FL 33612, USA; 7 Department of Urology, The Netherlands Cancer Institute, Antoni van Leeuwenhoek Hospital, 1066 CX Amsterdam, The Netherlands; 8 Department of Urology, University of Texas MD Anderson Cancer Center, Houston, TX 77030, USA; 9 Division of Medical Oncology, Department of Oncology, Mayo Clinic, Rochester, MN 55902, USA; 10 Department of Urology, IRCCS San Raffaele Hospital and Scientific Institute, 20132 Milano, Italy; 11 Department of Urology, University Medical Center Rostock, 18051 Rostock, Germany

We read with great interest the manuscript by Brassetti et al. recently published in your journal and hope it will encourage discussion and debate around the optimization of the surgical management of patients with penile cancer (PECa) [1]. In recent years, various scores have been established to assess postsurgical outcomes for other urological tumor types [2,3,4]. Brassetti et al. have now made the first attempt to summarize the quality of surgically treated PECa by using an overall score with their Tetrafecta criteria. A multicenter study cohort of 154 patients with surgically treated PECa, in clinical stages I–IIIB (American Joint Committee on Cancer/AJCC) was examined to determine the proportion that met the four criteria: (1) negative surgical margin (NSM), (2) no complications in Clavien–Dindo grades 3+ (< CDG-3), (3) a minimum number of excised inguinal lymph nodes (ILN), 7 per groin, and 4) no evidence of disease within 12 months of surgery (NED-12 months). The individual criteria were met by 92%, 73%, 65%, and 76%, respectively, resulting in a proportion of 29% (45/154) that achieved all the Tetrafecta criteria. Age was the only factor that independently predicted the presence of all four criteria, with each additional year of life reducing the likelihood of a Tetrafecta outcome by a relative 3% (95% confidence interval: 1–6%). PECa patients with a Tetrafecta outcome showed lower all-cause mortality (ACM) after 2 years (21 vs. 42%) and after 5 years (24 vs. 49%), compared to the PECa patients who did not meet all four criteria (*p* = 0.01).

**Are the Suggested Tetrafecta Criteria Correct and Robust?**

In their title, Brassetti et al. set out the aims of their Tetrafecta criteria; summarizing surgical quality indicators on the one hand and indications for satisfactory cancer control on the other. Whilst we applaud the authors for developing their concept, we do not believe they managed to fulfill their aims in full. In addition, it is not clear how they selected their four criteria. Regarding the quality indicators of the surgically-treated PECa, the selection of NSM and < CDG-3 seems quite reasonable. However, in our opinion, the criterion of at least 7 ILN per groin will potentially exclude cN0 patients treated safely and to a high quality with either dynamic sentinel node biopsy (DSNB) or modified ILN dissection (ILND), both of which are standardized procedures of surgical ILN staging, recommended in the European Association of Urology guidelines [5]. Furthermore, integrating a time-dependent criterion, as summarized by NED-12 months, into a score to assess the quality of the surgical therapy is not necessarily user-friendly, as it cannot be reported or used during the active treatment phase. Such a limitation was experienced with a Pentafecta score, developed in 2015 for optimizing outcomes after radical cystectomy, which also included a 12-month follow-up assessment [4].

Furthermore, we consider the presented Tetrafecta score to be potentially unsuitable in predicting ACM after surgical therapy for PECa. Brassetti et al. were able to demonstrate a certain discriminative quality for the aggregated score, although there are a plethora of better-designed scores for this endpoint, which have also been validated in this regard [6,7]. It is very likely that NSM predicts the endpoint and, eventually, it is also possible that there is an independent contribution from the number of ILN removed (especially when this results in the removal of micrometastases in the ILN). However, it is very unlikely that the < CDG-3 criterion impacts ACM (whereby we want to exclude a death caused by the surgical intervention of course). In addition, the inclusion of the time-dependent endpoint, NED-12 months, for the prediction of the time-dependent endpoint of ACM after, for example, 24 months is highly problematic because, although there is most likely a very close correlation here, there is also likely to be a considerable “circular reasoning fallacy”. We also believe three separate steps are necessary to establish whether the Tetrafecta score predicts ACM: (1) investigation of the individual contributions of each of the four criteria on the ACM endpoint, (2) specification of the predictive accuracy of the Tetrafecta score in ACM prediction, and (3) the simultaneous inclusion of the Tetrafecta score and, for example, the pathologic AJCC staging in one multivariate model to show that the Tetrafecta score has at least an independent influence on ACM prediction.

In summary, it is not clear to us on what basis the four criteria were selected (was there a moderated decision-making process among PECa experts as with the named Pentafecta score for radical cystectomy [4] or a moderated Delphi method?), whether they are user-friendly, and we do not believe they achieve the ultimate goal of high-quality care. To illustrate, let us consider a virtual patient with PECa stage pT1b (restricted to the glans penis). If the patient undergoes a partial penectomy and a radical ILND with 7 plus 7 ILNs (7 ILNs per groin) removed and remains complication-free and without disease recurrence within 12 months, he fulfills the Tetrafecta outcome. Conversely, if the patient undergoes organ-preserving surgery (e.g., glansectomy) and a DSNB, they do not achieve the Tetrafecta outcome. In both cases, the ACM will not differ, yet in our opinion, the first treatment option represents an inferior therapeutic intervention for the patient due to its potentially greater impact on their quality of life, yet unlike the second option, it fully satisfies the Tetrafecta criteria.

**The Effect of Tumor Stage and Comorbidities on Achieving the Tetrafecta Outcome**

The NED-12 months criterion within the Tetrafecta score is not exclusively dependent on the quality of the surgical treatment, although it is also significantly influenced by the tumor stage, the individual tumor biology, and any (neo)adjuvant oncological treatment that the patient receives. As a result, it is surprising that the pathological AJCC stage (which indicates whether a patient is pN+ and to what extent) did not appear to influence the Tetrafecta outcome. Of those that achieved the Tetrafecta criteria, 27% were pathological AJCC stage 3 and 38% were stage 4. This compares to the 29% who were stage 3 and 36% who were stage 4, in those who failed to meet the Tetrafecta outcome. In this context, it would be interesting to know what proportion of patients received multimodal treatment (in particular, how many patients with pN3 received neoadjuvant or adjuvant chemotherapy). It also highlights that high-quality PECa surgery, as expressed by the two other oncologically-relevant Tetrafecta criteria (i.e., extensive, and thorough ILND and the mandatory achievement of NSM) is achievable in more aggressive PeCa and that they are of great importance for a satisfactory oncological outcome.

It would also be interesting to see whether the achievement of the Tetrafecta outcome affects cancer-specific mortality (the analysis of this oncologic endpoint is mentioned by the authors in the Materials and Methods, although is not addressed again in the results section of the manuscript). To help interpret the survival data, we would also like to know the median follow-up (which unfortunately was also not provided).

It is important to reflect that none of the 24 patients with an ASA (American Society of Anesthesiologists) score of 3+ achieved the Tetrafecta outcome. Partial penectomy or organ-preserving treatment can be successfully performed under a local anesthetic penile block, thus, we wonder whether it was an inability to achieve an adequate ILND or a higher complication rate in this group, which led to this finding. Whilst the risks and benefits of intervention must be considered in every case, we would recommend adequate invasive nodal staging and treatment in higher-risk surgical patients, where possible.

**The Way Forward**

In our view, the Tetrafecta score reported by Brassetti et al. represents an important first step toward quality assurance of primary surgical therapies for PECa. However, due to its hybrid nature, as described above, it does not fully achieve this noteworthy goal.

With the objective of optimizing the Tetrafecta score, we (that are 13 authors) defined 13 criteria for PECa patients in clinical AJCC stages 1 to 4 (T1-3N0-3, but M0) in a moderated selection process (modified Delphi method with a total of four rounds), within the working group. To this end, three out of the four criteria established by Brassetti et al. were used with only minor modifications (C1–C3), and ten additional criteria were added to these criteria (thus, C1 to C13 were available for selection, Figure 1). The strongest discussion within our group was regarding the integration of a minimum surgical case volume of treated PECa into these 13 criteria, where a required annual case volume of at least 15 per hospital was finally defined (C13). This threshold was the lowest common denominator that our group could agree on, although clearly higher thresholds (e.g., 40+) are desirable as they are supported by the literature and also by recommendations from the National Institute for Health and Care Excellence (NICE) and eUROGEN [8,9,10,11]. On one hand, even this threshold value chosen by us excludes ambitious departments with a lower-case volume of PECa from fulfilling the complete Pentafecta score, although on the other hand, the author group agreed that a higher case volume would result in positive outcomes for patients, which may not be immediately apparent and may only manifest in the medium to long term. Each of the 13 PECa experts was then allowed to select five criteria for their individual Pentafecta score through a secret individual vote (Figure 1). Afterward, the primary 13 criteria were aggregated and evaluated, and those 5 criteria with the highest number of ratings were combined in the final Pentafecta score.

Of importance, the final Pentafecta score that we developed included three criteria that are achieved too infrequently by PECa patients in AJCC clinical stages 1 to 4, despite the various guideline recommendations [5,12]. Regarding these three criteria (C4–C6), the final Pentafecta score clearly calls for (1) the performance of organ-preserving surgical therapy with the goal of NSM, as far as the tumor stage allows (< pT3), (2) ILND should be performed from tumor stage T1G2N0 (preferably as DSNB or as modified ILND in cN0), and (3) the patient should be discussed by an interdisciplinary tumor board and offered the options of perioperative chemotherapy (neoadjuvant in cN3 and possibly in T4, adjuvant in pN2-3) (Figure 1).

Furthermore, it is interesting that criterion C2 (< CDG-3) did not make it into the final Pentafecta score, nor did any of the other Tetrafecta criteria. In contrast, a 3-month maximum time interval between surgical therapy of the primary tumor and ILND (C8) was considered an important quality standard to be met for an advised Pentafecta outcome [13]. The minimum annual caseload of 15 surgically treated PECa patients also achieved it in the Pentafecta score (C13). However, it is important to us that lower-volume departments can also evaluate their outcome with respect to the Pentafecta criteria. In such circumstances, we suggest a combined notation for outcome according to the Pentafecta criteria based on the number of total Pentafecta criteria met, followed by the number of Pentafecta criteria fulfilled without criterion C13 in parentheses. Thus, the outcome of a patient meeting all but the clinic caseload criteria would be reported as 4 (4). When using the Pentafecta score, it should also be noted that not every one of the criteria is appropriate for every patient. We recommend that criteria that do not apply in an individual case are counted as if they have been achieved. For example, clinical AJCC stage 1 includes patients with pT1G1 tumors who do not require ILND or multimodal treatment (perioperative chemotherapy). In these patients, criteria C5, C6, and C8 will not count but should be evaluated positively.

In conclusion, we believe that Brassetti et al. have performed an excellent job highlighting the key issues and considerations in surgical treatment for PECa, and we look forward to future research in this area. Of course, we hope that this letter, as well as our newly developed Pentafecta score, will stimulate further discussion and debate on this important topic and that it will help to ensure that PECa patients receive the highest quality of surgical and oncological care possible. We also believe that the Pentafecta score generated by expert consensus can provide quality assurance with regard to PeCa surgery and management strategies in urologic departments and we welcome the urologic community to help validate the Pentafecta score with regard to patient-relevant and patient-reported outcomes.

## Figures and Tables

**Figure 1:**
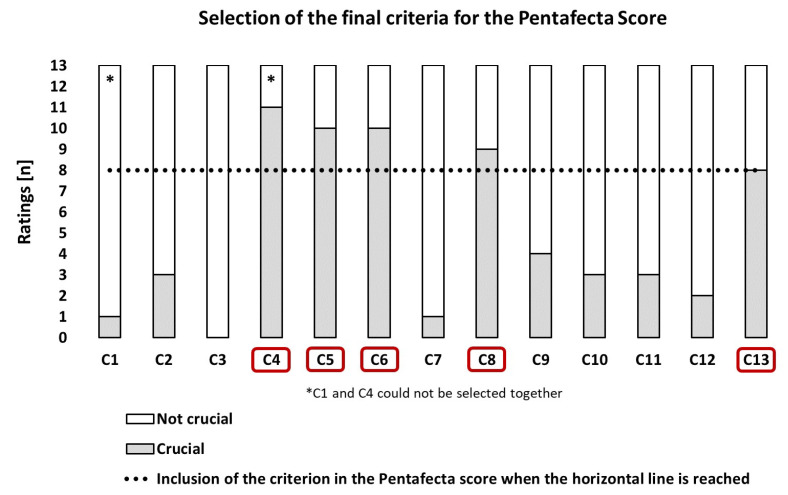
Formation of the final Pentafecta score based on the ratings of 13 experts on 13 predefined criteria (C1–C13). Legend: **C1:** There are no specifications for surgical primary therapy—achieving a negative surgical margin is the sole treatment goal. **C2:** There should be no postsurgical complications of severity grade 3+ (according to Clavien–Dindo) occurring within a time interval of 3 months after surgical therapy of the primary tumor and after nodal lymph node staging. **C3:** If indicated, the bilateral inguinal lymph node dissection should always include a minimum number of 14 removed lymph nodes. **C4:** Organ-preserving surgery of the primary tumor is performed if the tumor stage allows (< pT3), although always with the aim of achieving a negative surgical margin. **C5:** Starting at the tumor stage pT1G2N0, a bilateral inguinal lymph node staging should be performed in all patients, using modified inguinal lymph node dissection (ILND) or dynamic sentinel node biopsy for N0, and radical ILND for N1–2. **C6:** There should be access to an interdisciplinary tumor board for expert advice and discussion on perioperative chemotherapy (neoadjuvant/adjuvant) for patients with fixed inguinal lymph node metastases (cN3) and patients with pN2–3 after surgical lymph node dissection. **C7:** The treating hospital should have the interdisciplinary option for radiotherapy (EBRT/Brachytherapy) and should also present this treatment option to their patients in clinical tumor stage T1–2 with a tumor size < 4 cm. **C8:** If indicated, the inguinal lymph node dissection should be performed within a time window of 3 months for the surgical therapy of the primary tumor. **C9:** Every patient undergoing surgical primary therapy for penile cancer should receive a 5-year comprehensive individual oncological follow-up (aftercare) plan and be offered the possibility of psychological counseling or treatment, regardless of the extent of the procedure. **C10:** Surgery for the primary tumor should always be performed with frozen section analysis of the margins. **C11:** Refer the patient to a specialist lymphoedema therapist (physiotherapist) following ILND surgery. **C12:** Both surgical interventions on the primary tumor and potential ILND should be performed in accordance with current guideline recommendations for antibiotic prophylaxis. **C13:** The surgical primary therapy (primary tumor and, if necessary, lymph nodes) should always be carried out at a center with a minimum case volume of *n* = 15 primary treated cases of penile cancer per year.
